# Di (2-ethylhexyl) Phthalate Exposure Impairs the microRNAs Expression Profile During Primordial Follicle Assembly

**DOI:** 10.3389/fendo.2019.00877

**Published:** 2019-12-13

**Authors:** Jiao-Na Zhang, Rui-Qian Zhang, Jing-Cai Liu, Lan Li, Wei Shen, Xiao-Feng Sun

**Affiliations:** Key Laboratory of Animal Reproduction and Germplasm Enhancement in Universities of Shandong, College of Life Sciences, Qingdao Agricultural University, Qingdao, China

**Keywords:** DEHP, miRNAs-seq, miRNA expression profile, primordial follicle assembly, mouse

## Abstract

This research was performed to estimate the potential effects of Di (2-ethylhexyl) phthalate (DEHP) on changes of ovarian miRNA expression profile during mouse primordial follicle assembly using miRNAs-seq analysis. The ovaries of newborn mice were collected and *in vitro* cultured with different concentration of DEHP for 72 h. Then they were prepared for miRNAs-seq analysis. The results indicated that DEHP exposure altered ovarian miRNA expression profile of newborn mice. Eighteen differentially expressed miRNAs were screened after 100 μM DEHP exposure. The target mRNAs of differentially expressed miRNAs were predicted and further analyzed through gene ontology (GO) enrichment analysis and pathway enrichment analysis. Our results showed that the differentially expressed miRNAs from DEHP exposure can regulate ovarian development by targeting mRNAs involved in MAPK, mTOR, FoxO signaling pathways. Three miRNAs of miR-32-5p, miR-19a-3p, and miR-141-3p were randomly selected from the differentially expressed miRNAs to quantify their expression level by miRNA qRT-PCR. The results of qRT-PCR and miRNA-seq were consistent. Considering one of its target gene PTEN of miR-19a-3p and the decreased level of pAKT and increased Bax/Bcl-2 under DEHP exposure, we speculated that the altered expression of miR-19a-3p by DEHP exposure affected mouse primordial follicle assembly via PI3K/AKT1/mTOR signaling pathway. Epigenetic changes are one of the most important targets of toxicant exposure. The effects of DEHP exposure on microRNA (one of the epigenetic regulators) expression profile were uncovered to enrich the research on relationship of epigenetics and toxicant exposure.

## Introduction

Di (2-ethylhexyl) phthalate (DEHP) is one of the most common phthalates widely used in plastics (1) for medical apparatus and instrument, food packaging, and plastic drink bottles (2). Unfortunately, DEHP can leach out from the plastic and have become one of the common environmental contaminants (2). To human, diet is the largest DEHP exposure source (3), especially for fast food, perhaps because of the massive use of plastic packaging or disposable PVC gloves during fast food preparation (4). Medical procedure is another exposure source for patients and some of neonates (3, 5). Kavlock et al. compared published data on blood DEHP level before and after medical procedures including cardiac bypass and exchange transfusion to adult, surgery, and platelet transfusion to infant, which found that most medical procedures promoted the blood DEHP level (3). So human can be widely exposed to DEHP. It has been proved that DEHP is the most severe reproductive toxicant among all the phthalates by comparison of seven phthalates using continuous breeding protocol (6). Though it quickly metabolizes to its active metabolite mono (2-ethylhexyl) phthalate (MEHP), when it enters the animal body, detectable DEHP level also remains in the plasma and peritoneal fluid (3, 7). In 2001, a study was performed in 42 Swedish primipara to detect DEHP and its metabolites in the samples of breast milk, blood or serum, and urine, which showed that the concentration of DEHP in the samples of milk was from 0.45 to 305 ng/mL and in blood from 0.5 to 129 ng/mL (8). And DEHP itself has been proved to target reproductive organ/tissue *in vivo* and *in vitro*. DEHP as an endocrine disrupting chemicals (EDC) brings reproductive toxicity to both male and female human and wild animals (3). In male, DEHP could decrease testis weight and volume, lower the functions of sperm (9), and increase the percentage of abnormal sperm and enhance the DNA damage level of sperm (10). In female, it has been reported that DEHP exposure affects germ cells development at various stages. DEHP could influence oocyte meiosis during *in vitro* culture of 12.5 dpc (days post-coitum) female mouse genital ridge (5). DEHP exposure at a dosage of 20 and 40 μg/kg by intraperitoneal injection caused a lower proportion of antral follicles with a diameter >150 μm (11). DEHP also decreased the weight of ovary and uterus and promoted the quantity of follicles (1).

Ovary is a primary organ regulating the reproductive and endocrine functions of female animals (12). DEHP and its metabolites were able to be detected in amniotic fluid samples and ovarian follicular fluid, which gave the evidence that DEHP could reach ovaries and play roles there (13). Gestational days 0–3 of mouse is the important development stage for cyst breakdown and primordial follicle assembly (12). The foundation of the female animals' reproductive competency is to establish the primordial follicle pool, because it determines the fecundity of a female animal throughout the reproductive life (14). So that any defects in the process can bring negative impact on female animal reproductive potential.

MicroRNAs, endogenous, small, non-coding single-stranded RNA molecules, post-transcriptionally regulate the expression level of genes involved in various physiological and pathological processes, such as cell proliferation, differentiation, cell death, hormone biosynthesis, and secretion (15) via base pairing of the 3′-untranslated regions (3′-UTRs) of their target mRNAs. The development of the mammalian ovary is high dynamic, which is regulated by coordinated expression of plenty of genes in a spatially and temporally specific manner (16). Moreover, post-transcriptional regulation is as important as transcriptional regulation in controlling gene expression. As one of the most important epigenetic regulators, miRNA has been proved expressed in ovaries regulating granulosa cells proliferation, oocyte maturation, and reproductive hormone secretion, thereby regulating ovary function and ovarian disorder (17–19). Moreover, changes of epigenetics are the dominant reasons that animals regulate their gene expression in response to environmental factors such as toxicant exposure (20). So that the epigenetic changes effected by environmental toxicant are now becoming the focus of research. Ovary cyst breakdown and primordial follicle assembly also highly rely on specific genes expression (21). Though there is evidence that phthalate MEHP can alter the expression of the specific oxidative stress responsive miRNAs (22), the changes of whole miRNAs profile and the underlying mechanism needs to be further explored. In this study, we aimed to uncover the changes of miRNA profile after DEHP exposure, their roles and the mechanisms underlying the course of cyst breakdown and primordial follicle assembly.

## Materials and Methods

### DEHP

DEHP (Sigma-Aldrich-36735) with the purity ≥99.7% used in this project was from Sigma-Aldrich company (St. Louis, MO), then dissolved in DMSO and diluted to the final concentrations of 10 μM (3.9 μg/ml) and 100 μM (39 μg/ml) with culture medium. The detailed DEHP dilution flowchart was shown in Figure S1. In the working solution, the concentration of DMSO was 1.57*10^−4^ μl/400 μl/well.

### Animals

CD1 mice used in the project were obtained from Vital River Laboratory Animal Technology Co. Ltd. (Beijing, China). Pregnant mice of 6–8 weeks old were maintained at 21–22°C. After given birth, 297 female newborn mice were selected and the ovaries were collected, randomly divided into three groups and cultured *in vitro* with DEHP at the concentration of 0 μM (vehicle control DMSO), 10 and 100 μM, respectively, for 72 h. In each group, every 2–3 ovaries were cultured in a well of 24-well tissue culture treated plates at 37°C. The culture medium used for ovaries culture were Dulbecco's modified Eagle's medium/F12 (DMEM/F12) (HyClone, SH30023.01B, Beijing, China) and a-minimal essential medium (a-MEM) (HyClone, SH30265.01B, Beijing, China) (1:1) supplemented with 10% FBS (Gibco, 10099-141, USA), 0.23 mM sodium pyruvate (Hyclone, SH40003-12, Beijing, China), 100 IU/ml of penicillin G, and 100 mg/ml of streptomycin sulfate (penicillin- streptomycin solution, HyClone, SV30010, Beijing, China). When completing the culture, 360 ovaries (120/group) from 180 mice were used for miRNAs-seq analysis. Ninety ovaries (30/group) from 45 mice were used for miRNA qRT-PCR. Ninety ovaries (30/group) from 45 mice were used for western blotting and 54 ovaries (18/group) from 27 mice were used for immunofluorescence staining and TUNEL staining. All the animal procedures were examined and approved by the Ethics Committee of Qingdao Agricultural University.

### Immunofluorescence Staining

After DEHP exposure *in vitro*, the ovaries were collected to be used for immunofluorescence staining according to the standard protocol. Briefly, after fixed overnight with 4% paraformaldehyde solution, the ovarian sample was paraffin-embedded and serially sectioned into 5 μm sections. For each ovaries, about 30 serial sections were obtained. Then we used xylene to remove paraffin and tissue rehydration was performed with gradient alcohols. Trisodium citrate were heated for antigen retrieval under the condition of 96°C for 10 min. And then the non-specific binding was removed by blocking for 45 min with blocking solution (3% BSA and 10% goat serum dissolved in TBS). To quantify the germ cells and follicles, the germ cell specific marker MVH was used to label germ cells. Briefly, the sections were firstly incubated with polyclonal antibody of rabbit-derived anti-MVH (Abcam, ab13840) diluted in blocking solution at 1:150 for 8 h at 4°C, and were then treated with FITC/Cy3 labeled goat anti-rabbit IgG (1:60 diluted, Beyotime, A0562/A0516, Nantong, China) at 37°C, 40 min. Hoechst33342 (Sigma, B2261, USA) were used for section incubation to locate cell nucleus. Fluorescent images were then examined and photographed using a fluorescent microscope (Olympus, BX51, Japan). Germ cells in follicles or within cysts were distinguished and counted, respectively. Among all of the sections of one ovary, one out of every four serial sections was selected for counting germ cells.

### TUNEL Staining

After immunofluorescence stained with MVH antibody and FITC labeled secondary antibody, proteinase K was used for treatment the sections for 25 min. Then we used TUNEL BrightRed Apoptosis Detection Kit (Vazyme, A113, Nanjing, China) to display the apoptotic cells and to evaluate the number and the rate of the apoptotic cells in ovaries complying with the manufacturer's protocol. Then the slides were incubated with TUNEL reaction mixture for 60 min at 37°C away from light. Ultimately, after stained by Hoechst33342, they were examined and photographed under a confocal laser microscope (Leica TCS SP5II, Germany).

### RNA Extraction, Reverse Transcription, and RNA-Seq

After treatment with DEHP, the ovaries were homogenized in TRIZOL to extract total RNA following the manufacturer's protocols. Then they were send to sequence using HiSeq2500 sequencer at Novogene Bioinformatics Technology Co., Ltd (Beijing, China). Bioinformatic analyses of the data were then performed according the workflow described previously (23, 24).

### miRNA-Seq Data Preprocessing and Analysis of Differential Expression

To obtain high quality sequencing data, the raw data were treated to cut the linker sequences, remove the low-quality sequences and trim off the sequences for quality control. The treated reads were then compared with the mouse reference genome using the software of bowtie2. The expression level of miRNAs were quantitatively analyzed using MiRDeep2 software and the differentially expressed miRNAs were confirmed.

### Analyzing Differentially Expressed miRNAs

R package DESeq2 was used to normalize the expression level of miRNAs. The miRNAs with expression level of |log2FoldChange|>2 and FDR <0.05 were regard as differentially expressed miRNAs. The classical Hypergeometric distribution tests were used to identify differentially expressed miRNAs pairwise comparison in three concentration groups of 0, 10, and 100 μM. The raw *p*-value into the false discovery rate (FDR) was corrected by the method of Benjamini et al. (25).

### Predicting miRNA Target Genes and Analyzing Differentially Expressed miRNAs-Target Genes Interactions

The online software of miRWalk2.0 (http://zmf.umm.uni-heidelberg.de/apps/zmf/mirwalk2) was applied to our study to predict the target genes of differentially expressed miRNAs. The interactions of differentially expressed miRNAs-target genes were also analyzed by the online software miRWalk2.0. Finally, network of differentially expressed miRNAs-target genes was drawn and analyzed by cytoscape software.

### Gene Ontology (GO) Enrichment Analysis

According to Gene Ontology Database, the targets roles of the differentially expressed miRNAs between control and DEHP-exposed ovaries were analyzed by Gene ontology (GO) that is a common tool for analyzing large-scale genes functional enrichment (26). GO enrichment analysis was carried out by R package clusterProfiler and *P* < 0.05 and FDR < 0.1 was set as the cutoff criterion for GO enrichment analysis.

### Analyzing Pathway Enrichment

Kyoto Encyclopedia of Genes and Genomes (KEGG), a bioinformatics database commonly used in analyzing pathway enrichment of large-scale molecular datasets (27) was also used for pathway enrichment analysis in this study. R package clusterProfiler provides analytic methods to extract valuable biological information in a large-scale genes (28). We used R package clusterProfiler in this study for analyzing KEGG pathway enrichment of targets for differentially expressed miRNAs. Pathways screened was analyzed in depth for significant differences based on the cutoff criterion of *P* < 0.05 and FDR < 0.1 for KEGG enrichment analysis.

### Small RNA Extraction and miRNA qRT-PCR

To confirm the results of miRNA-seq, miR-32-5p, miR-19a-3p, and miR-141-3p were randomly selected from the differentially expressed miRNAs and quantified their expression levels. Small RNA was extracted and collected using TaKaRa RNAiso for Small RNA kit (Takara, 9753A). The corresponding primers (CD202-0093, CD202-0030, and CD202-0112) were purchased from Tiangen Biotech (Bingjing, China) Co. Ltd. miRNA first strand synthesis and quantification were carried out by a miR-X^TM^ miRNA first strand synthesis and SYBR qRT-PCR kit (Takara, 638313, 638314). Each reactions were carried out with 2 μl cDNA, 10 μl of SYBR Advantage Premix, 1 μl miR-specific primers (5 μM), 1 μl mRQ 3′primer and 6 μl of RNA-free water, at 95°C for 6 s, then 45 cycles of 95°C for 6 s, 60°C for 20 s, finally cooled at 4°C in a LightCycler 480 real-time PCR instrument (Roche LC480). U6 was applied for normalizing the expression level of each miRNA. The relative amount of expression for each miRNA was calculated using 2^∧^-(target miRNA CT value – U6 CT value). Each amplification was carried out in triplicate.

### Western Blotting

After 72 h DEHP administration, ovaries were collected for western blotting analysis. The proteins from each groups were homogenized and extracted using RIPA lysis buffer (29, 30). Then the proteins were separated by running SDS page gel and electrophoretically transferred to PVDF membranes. Blocked with 5% BSA for 2 h, the membranes were incubated overnight using the primary antibody at concentration of 1.0 μg/ml. The details of the primary antibodies are shown in Table 1. After washed with TBST for three times, HRP-conjugated goat anti-rabbit IgG (Beyotime, A0208, Shanghai, China) was then used to incubate the membranes at 37°C for 2 h at a dilution of 1:2,000 in TBST. The bands were visualized by chemiluminescent method using BeyoECL Plus kit (Beyotime, P0018S, Shanghai, China). The specific band intensities were digitally quantified using Actin as internal reference with alpha view software. Three independent experiments were performed using samples treated in different time.

**Table 1:** Primary antibodies used in this study.

**Antibody**	**Company**	**Catalog**
Anti-Actin rabbit polyclonal antibody	Sangon	D110001
Anti-PTEN rabbit polyclonal antibody	Sangon	D261095
Rabbit anti-pAKT antibody	Abcam	Ab66138
Rabbit anti-Bax antibody	Cell signaling	#2772S
Rabbit anti-Bcl-2 antibody	Beyotime	AB112

### Statistical Methods

All data were analyzed with GraphPad Prism software and were represent as the mean ± SD of at least three independent experiments. Statistical differences between each groups were analyzed and determined by a one-way analysis of variance (ANOVA) followed by the Tukey's test. The results were regarded as significance when *P* < 0.05.

## Results

### DEHP Exposure Affects Germ Cell Cyst Breakdown and Primordial Follicle Assembly

**Figure 1:**
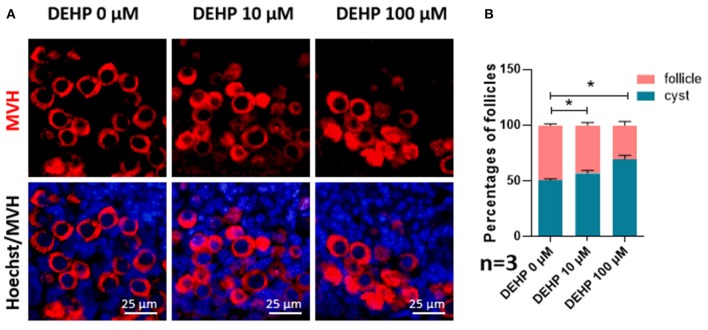
DEHP Exposure affects germ cell cyst breakdown and primordial follicle assembly. **(A)** Histological sections of 3 dpp mouse ovaries cultured with different concentration of DEHP for 3 days. Oocytes are stained red with anti-Mvh antibody, nuclei are stained blue with Hochest 33342; **(B)** percent of germ cells in nests and follicles after treated with different concentration of DEHP. The results are presented as mean ± SD of at least three independent experiments. *P < 0.05.

For detecting the impacts of DEHP on germ cell cyst breakdown and primordial follicle assembly, immunofluorescence staining was performed. After MVH immunofluorescence staining, germ cells in follicles or within cysts can be distinguished clearly. From Figure 1, both 10 and 100 μM DEHP treatment significantly decreased the percentage of primordial follicle. The percentage of cysts increased from 50.71 to 69.80% after 100 μM DEHP exposure. Accordingly, the percentage of primordial follicles decreased from 49.29 to 30.20% after 100 μM DEHP exposure. And the differences were significant.

### DEHP Induces Cell Apoptosis

**Figure 2:**
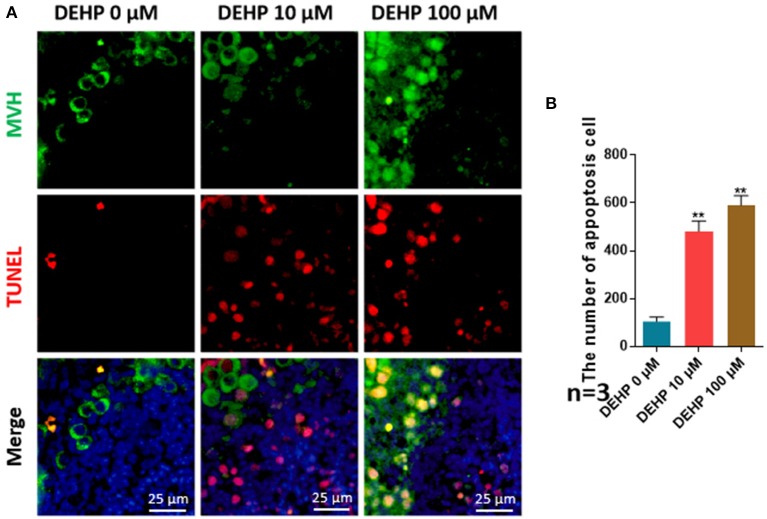
DEHP induces the cell apoptosis. **(A)** Histological sections with TUNEL stainning. Oocytes are stained green with anti-Mvh antibody, apoptosis cells are marked red with TUNEL, nuclei are stained blue with Hochest 33342; **(B)** percentages of TUNEL positive oocytes. The results are presented as mean ± SD of at least three independent experiments. **P < 0.01.

The number of apoptotic cells was significantly promoted by DEHP treatment (10 μM vs. control 481.444:104.778; 100 μM vs. control 590.083:104.778), which rose more than four and five times after 10 and 100 μM DEHP exposure compared with that of control (Figures 2A,B). Moreover, the apoptosis occurs not only in somatic cells but in germ cell by merging the MVH signaling and TUNEL signaling (Figure 2A). Especially for the 100 μM DEHP treated ovaries, the apoptotic germ cells had a remarkable increase (Figure 2A).

### DEHP Exposure Alters the miRNA Expression Profile in the Ovaries of Newborn Mice

**Figure 3:**
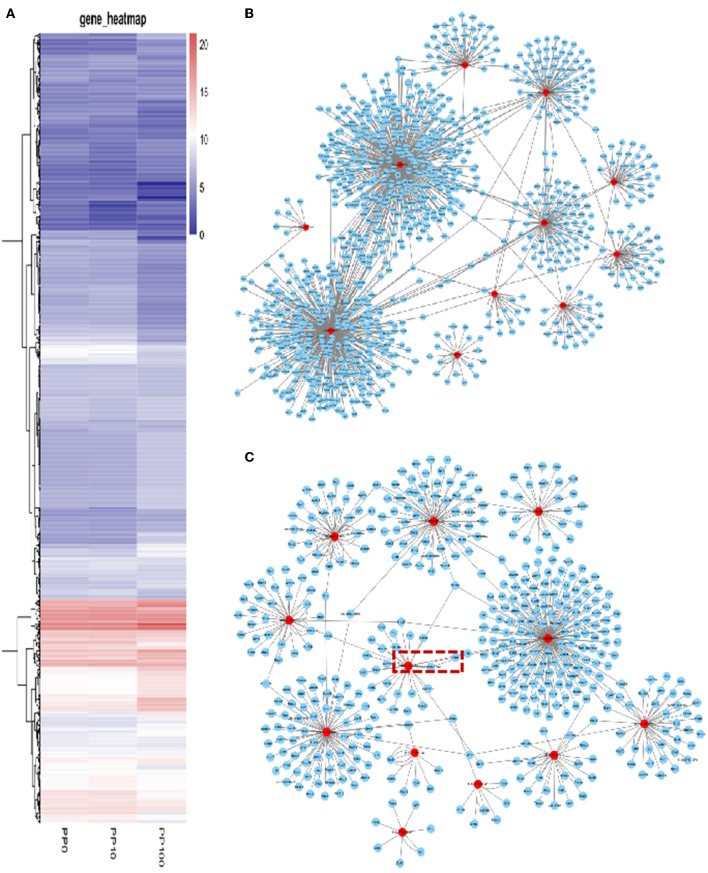
DEHP Exposure Alters the miRNA Expression profile. **(A)** Heatmap shows the Euclidean distances between the samples calculated from the regularized log transformation; **(B,C)** predicted target genes of differentially expressed miRNAs.

To study the effects of DEHP on the expression of miRNA in the ovaries of newborn mice and on the development of murine ovaries, ovaries from newborn mice were cultured *in vitro* with DEHP at the concentration of 0, 10, and 100 μM for 72 h. At the end of the culture, miRNA-seq was used to obtain the differentially expressed miRNAs caused by exposure of DEHP. Cluster analysis of miRNAs expression in different concentration of DEHP (0, 10, and 100 μM) showed that there was little difference between the groups of 10 and 0 μM DEHP in the expression of miRNAs, but significant difference between the groups of 100 and 0 μM DEHP in the expression of miRNAs. And the expression of miRNAs was significantly different between 100 μM DEHP group and 10 μM DEHP group (Figure 3A). On the basis of the research criterion FDR < 0.05, a sum of 18 differentially expressed miRNAs were screened after 100 μM DEHP treatment, among which nine were upregulated and nine were downregulated (Table 2). And 19 differentially expressed miRNAs were screened between 100 μM DEHP treatment group and 10 μM DEHP treatment group, among which 12 were upregulated and 7 were downregulated (Table 3).

**Table 2:** Differentially expressed miRNAs between 100 and 0 μM DEHP treatment.

**miRNA ID**	**BaseMean**	**Log2FoldChange**	***P*-value**
MIMAT0000654	486.5502	−2.20312	0.011832
MIMAT0003498	15039.41	2.252842	0.012357
MIMAT0000249	13022.38	2.232852	0.012378
MIMAT0004661	3635.301	2.245133	0.012388
MIMAT0000651	343.2621	−2.2034	0.013399
MIMAT0000667	13.07698	−2.19936	0.014831
MIMAT0000674	407.1078	−2.12033	0.016099
MIMAT0000248	14022.13	−2.08197	0.016114
MIMAT0009458	8630.309	2.108539	0.018656
MIMAT0000153	80.54971	−2.12265	0.018717
MIMAT0017008	142.7016	−2.04089	0.020241
MIMAT0028043	34.49346	2.07605	0.020707
MIMAT0017276	429.6647	2.032281	0.021132
MIMAT0000516	874169.3	2.051925	0.021715
MIMAT0003476	33.30141	−2.06243	0.021831
MIMAT0003494	189.6363	2.010112	0.024296
MIMAT0004619	347.6832	2.002768	0.024429
MIMAT0000382	992.1017	−1.96979	0.021964

**Table 3:** Differentially expressed miRNAs between 100 and 10 μM DEHP treatment.

**miRNA ID**	**BaseMean**	**Log2FoldChange**	***p*-value**
MIMAT0004521	228.3168	2.590219	0.003921
MIMAT0003498	15039.41	2.393251	0.007869
MIMAT0009458	8630.309	2.309873	0.009968
MIMAT0000516	739.0609	2.215568	0.011968
MIMAT0004625	739.0609	2.215568	0.011968
MIMAT0000153	80.54971	−2.26536	0.012101
MIMAT0004661	3635.301	2.248865	0.012244
MIMAT0000651	343.2621	−2.19407	0.013798
MIMAT0019341	28.95159	−2.21454	0.01401
MIMAT0004846	122614.9	2.177046	0.014495
MIMAT0004932	9155.946	2.125907	0.016453
MIMAT0003494	189.6363	2.129889	0.017018
MIMAT0000137	4996.483	−2.04539	0.018659
MIMAT0004619	347.6832	2.091821	0.018762
MIMAT0014801	223.2425	2.089137	0.01898
MIMAT0000539	2766.481	−1.99261	0.020659
MIMAT0000674	407.1078	−2.0174	0.022301
MIMAT0000249	13022.38	2.022295	0.023492
MIMAT0001091	1206.7445	−1.97329	0.022577

### Prediction of the miRNAs Targets

miRNAs are endogenous small molecular RNAs, which can bind to its 3′-untranslated regions (3′-UTRs) and negatively control the expression of their target mRNAs. The prediction of differently expressed miRNAs targets is helpful for understanding the regulatory relationship between mRNAs and their target genes. miRWalk2.0 database was used for predicting the target mRNAs of differently expressed miRNAs. A total of 1,104 mRNAs were screened from 18 differentially expressed miRNAs between 100 and 0 μM DEHP treatment. And 537 mRNAs were screened from 19 differentially expressed miRNAs between 100 and 10 μM DEHP treatment.

Based on the multiple target mRNAs of miRNA, interactions between differentially expressed miRNAs and their target genes were analyzed, as well as the network of differentially expressed miRNAs and their targets (Figures 3B,C). Our results showed that MIMAT0000248, MIMAT0000382, and MIMAT0001091 can regulate hundreds of target mRNAs. Among them, PTEN is one of the targets of miR-19a-3p boxed in an open rectangle in Figure 3C. DEHP exposure changes the miRNAs profile then the expression level of their target mRNAs, thereby affecting the processing of ovarian development in the early stage.

### miRNAs Related Differentially Expressed Genes (DEGs) Involved in Gene Ontology (GO) Classification

**Figure 4:**
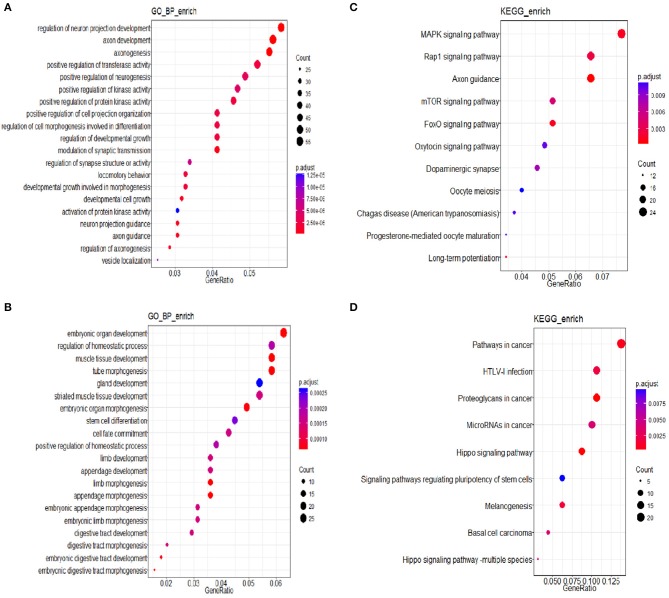
**(A,B)** miRNAs related differentially expressed genes (DEGs) involved in Gene Ontology (GO) Classification; **(C,D)** Kyoto Encyclopedia of Genes and Genomes (KEGG) pathways of miRNA related DEGs.

Three groups of differentially expressed genes were obtained by comparing every two groups among DEHP-exposed ovaries (0, 10, and 100 μM) from the data of miRNA-seq. Then based on the results of miRNAs related DEGs, the Venn diagram were constructed. Totally, 164 shared DEGs caused by 100 and 10 μM DEHP were detected. Then the DAVID was used to identify GO-enriched (*P* < 0.05 and FDR < 0.1) functions for the miRNAs related DEGs (Figures 4A,B). The DEGs from the ovaries treated with 100 and 0 μM DEHP were remarkably enriched in cell growth, cell development, and cell differentiation (Figure 4A). And the DEGs from the ovaries treated with 100 and 10 μM DEHP were significantly enriched in biological processes (Figure 4B).

### KEGG Pathways

The R package of clusterProfiler was applied to confirm the profound the Kyoto Encyclopedia of Genes and Genomes (KEGG) (*P* < 0.05 and FDR < 0.1) pathways to gain further insights into the roles of miRNA related DEGs. Eleven signaling pathways were detected by KEGG pathways analysis between 100 and 0 μM DEHP exposed ovaries, including MAPK signaling pathway, mTOR signaling pathway, FoxO signaling pathway, oocyte meiosis signaling pathway and progesterone-mediated oocyte maturation signaling pathway (Figure 4C; Table 4). Nine signaling pathways were detected by KEGG pathways analysis between 100 and 10 μM DEHP exposed ovaries, including Hippo signaling pathway, cancer related signaling pathway, and melanogenesis signaling pathway (Figure 4D; Table 5).

**Table 4:** KEGG through target genes of differentially expressed miRNAs between 100 and 0 μM DEHP treatment.

**KEGG ID**	**Description**	***p*-value**	***q*-value**	**Count**
mmu04360	Axon guidance	1.82E-06	0.00036	23
mmu04010	MAPK signaling pathway	1.22E-05	0.000967	27
mmu04068	FoxO signaling pathway	1.47E-05	0.000967	18
mmu04720	Long-term potentiation	2.61E-05	0.001293	12
mmu04015	Rap1 signaling pathway	4.84E-05	0.001914	23
mmu04150	mTOR signaling pathway	0.000108	0.003557	18
mmu04728	Dopaminergic synapse	0.000218	0.006159	16
mmu04921	Oxytocin signaling pathway	0.000333	0.00777	17
mmu04914	Progesterone-mediated oocyte maturation	0.000353	0.00777	12
Mmu05142	Chagas disease (American trypanosomiasis)	0.000405	0.008013	13
mmu04114	Oocyte meiosis	0.000479	0.008619	14

**Table 5:** KEGG through target genes of differentially expressed miRNAs between 100 and 10 μM DEHP treatment.

**KEGG ID**	**Description**	***p*-value**	***q*-value**	**Count**
mmu05205	Proteoglycans in cancer	5.62E-07	0.00010	17
mmu04390	Hippo signaling pathway	1.88E-06	0.00018	14
mmu05200	Pathways in cancer	8.50E-06	0.000543	22
mmu04916	Melanogenesis	2.57E-05	0.001229	10
mmu05166	HTLV-I infection	4.84E-05	0.001914	23
mmu05217	Basal cell carcinoma	9.42E-05	0.003007	7
mmu05206	MicroRNAs in cancer	0.000129	0.003182	16
mmu04392	Endometrial cancer	0.001507	0.032821	6
mmu04306	Hippo signaling pathway-multiple species	0.000133	0.003182	5
Mmu04550	Signaling pathways regulating pluripotency of stem cells	0.000364	0.007745	10

### DEHP Exposure Affects Primordial Follicle Assembly and Cell Apoptosis via AKT Pathway

**Figure 5:**
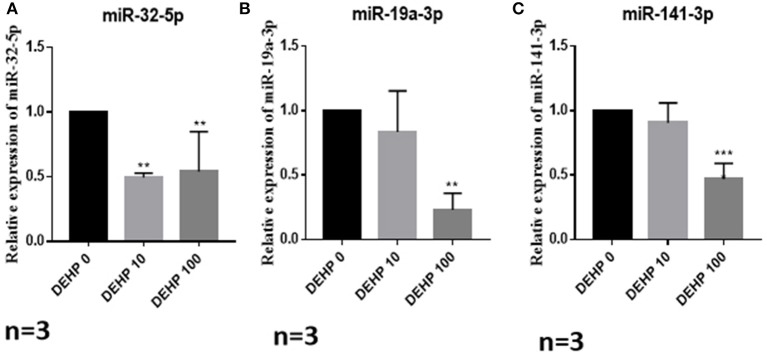
Verification of miRNAs expression by qRT-PCR. **(A)** miRNA expression of miR-32-5p, **(B)** miRNA expression of miR-19a-3p, **(C)** miRNA expression of miR-141-3p. The results are presented as mean ± SD of at least three independent experiments. **P < 0.01, ***P < 0.001.

**Figure 6:**
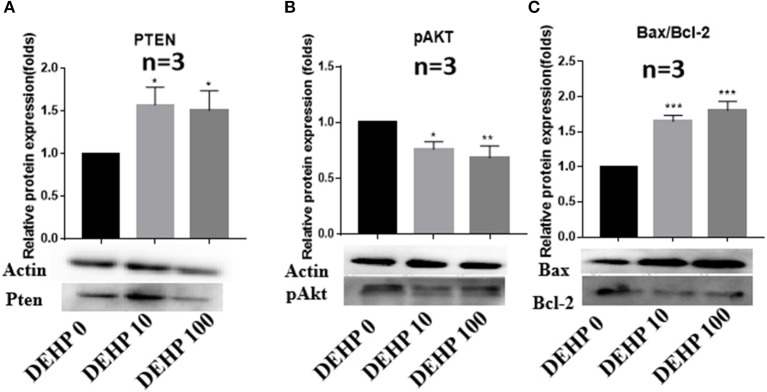
Verification of protein expression by western blot. **(A)** Western blot and the gray intensity of PTEN, **(B)** western blot and the gray intensity of pAKT, **(C)** western blot and the gray intensity of Bax and Bcl-2. The results are presented as mean ± SD of at least three independent experiments. *P < 0.05, **P < 0.01, ***P < 0.001.

To confirm the results of miRNA-seq, 3 miRNAs of miR-32-5p, miR-19a-3p, and miR-141-3p were randomly selected to quantify the expression level after DEHP treatment. The results of qRT-PCR displayed that the expression of miR-19a-3p and miR-141-3p were reduced by 100 μM DEHP, while, the level of miR-32-5p could be altered by both 10 and 100 μM DEHP (Figures 5A–C), which were similar to the results of miRNA-seq. Several molecules including AKT3, PTEN, and Pik3r3 in AKT signaling pathway were listed in the targets of differentially expressed miRNAs after predicting the targets of miRNAs. That's why we further detected several proteins expression involved in AKT pathway. WB results showed that the protein level of PTEN significantly increased (Figure 6A), and pAKT significantly decreased after DEHP exposure (Figure 6B). Meanwhile, the protein levels of Bax/Bcl2 usually representing the degree of cell apoptosis were detected increased after DEHP exposure (Figure 6C).

## Discussion

Generally, epidemiological study is the main approach to study the potential risks of toxicant exposure on human (31, 32), moreover, animal studies provide direct evidences to prove the specific harm and the underlying mechanisms. In research of toxicology, the selection of toxicant concentration is the key step of the whole experiment. The concentrations of DEHP used in this study were 10 μM (3.9 μg/ml) and 100 μM (39 μg/ml), which were based on our previously dose response test and the papers published by our research group (33, 34). Meanwhile, the maximum concentration used in our study (100 μM, 39 μg/ml) is within the widely accepted concentration range *in vitro* (35, 36). Phthalates are widely used in plastics industry to improve the flexibility of plastics. Several kinds of phthalates are reported to bring negative effects to animal development and reproductive system by inducing oxidative stress (37). Wang et al. reported that both DEHP (with the concentration of 25.6 μM) and its active metabolite MEHP (with the concentration of 35.9 and 359 μM) inhibited antral follicles growth of mouse ovaries by increased level of reactive oxygen species (35, 38). It is well-known that ROS (reactive oxygen species) can induce autophagy to protect cells from oxidative damage (39, 40). But excessive oxidative stress can induce cell apoptosis (41, 42). DEHP is one of the most abundantly used phthalates, which was quickly metabolized to its active metabolite MEHP, when it entered the animal body. MEHP has been detected to induce ROS-dependent cell autophagy through AKT1 pathway in human vascular endothelial cells (43). DEHP has also been proved to bring high ROS level in the ovaries of mice cultured *in vitro* by our research team (34). PI3K signaling pathways were the well-known targets of phthalates. It was found that DEHP exposure altered the estrous cyclicity and accelerated recruitment of the primordial follicle through PI3K signaling pathway in mice (13). Hannon et al. also proved that MEHP exposure accelerated mice folliculogenesis and inhibited mice steroidogenesis *in vitro* through PI3K signaling pathway (44).

Epigenetics focused on hereditable changes of gene expression without changes of DNA sequence. Epigenetic changes including DNA methylation, histone modification and non-coding RNAs are one of the most important targets of toxicant exposure to influence health and disease (45). Maternal exposure to DEHP changed the expression level of DNA methyltransferase of F1 fetal testes, further changed DNA methylation leading to the abnormal testicular function (46). It's suggested that oxidative stress responsive miRNAs miR-17-5p, miR-155-5p, and miR-126-3p could be promoted by MEHP in First Trimester Placental Cell Line HTR8/SVneo (22). This provides the evidence that phthalates alters the miRNAs expression, however the authors only detected three specific miRNAs. Changes of the whole miRNA profile in ovaries affected by phthalates are not well-understood. From our results, DEHP exposure altered the profile of ovarian miRNAs (Tables 2, 3). Gene ontology (GO) classification and KEGG pathways analysis (Figure 4, Tables 4, 5) were performed after predicting target genes of differentially expressed miRNAs. And the results showed that they were involved in MAPK and mTOR signaling pathways, which was consistent with the theory that PI3K/AKT1/mTOR signaling pathways were involved in cell autophagy and cell apoptosis (47). By transcriptome sequencing, Liu et al. also found a key gene in the *in vitro* cultured (0–3 dpp) mice ovaries named *Xdh*, which was related with oxidative stress induced by DEHP. *Xdh* gene knock down by RNAi could lower the oxidative stress and reduce cell apoptosis (34), which provided the evidence of effects of DEHP on oxidative stress and apoptosis by mRNAs sequencing.

**Figure 7:**
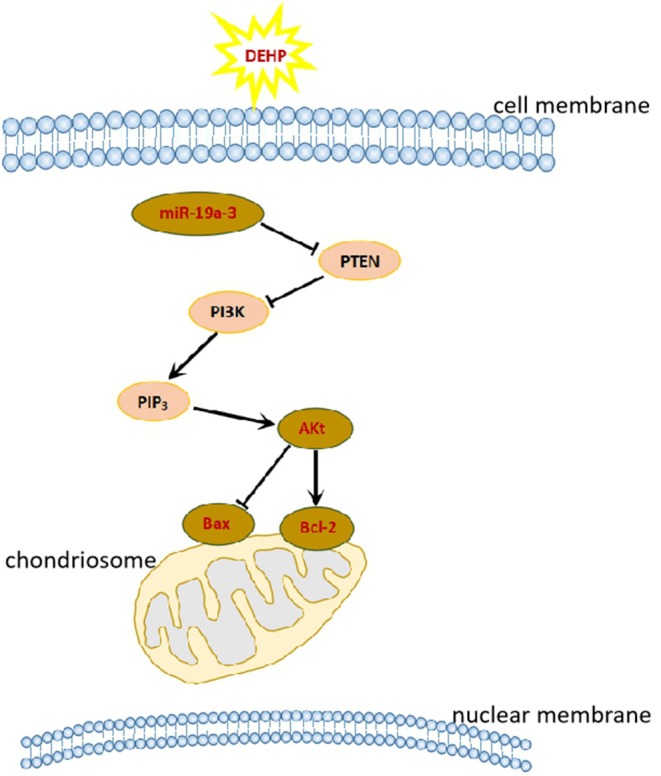
DEHP exposure affects primordial follicle assembly and cell apoptosis via PI3K/AKT signaling pathway.

In this study, we obtained the direct evidence that DEHP exposure altered the miRNAs expression profile by small RNA-Seq. And the target mRNAs of these differentially expressed miRNAs are involved in PI3K/AKT1/mTOR signaling pathways which associated with cell apoptosis. We can speculate that DEHP exposure alters the profile of mRNAs such as decreases miR-19a-3p, one of whose target is PTEN (Figure 3). Decreased miR-19a-3p by DEHP exposure, increases the level of PTEN, which decreases the phosphorylation of AKT. It is known that the accumulation of phosphorylated AKT in the cytoplasm of the cell can block the expression of Bax and induce the expression of Bcl-2, which inhibit the cell apoptosis by altering the permeability of mitochondrial membrane (Figure 7). In our study, besides decreased miR-19a-3p (Figure 5B), increased PTEN and decreased phosphorylated AKT level was also detected by WB after treatment with DEHP (Figures 6A,B). Furthermore, DEHP could significantly increase Bax/Bcl-2 at the protein level (Figure 6C), which was consistent with the results of TUNEL staining (Figure 2). The promoted apoptotic rate was probably induced by decreased level of phosphorylated AKT and miR-19a-3p after DEHP exposure. From our results, although there was no abvious difference in miR-19a-3p expression after 10 μM DEHP exposure, cell apoptosis and primordial follicle assembly were both detected significantly different between 10 μM DEHP exposure group and the control. We deduced that the slight changes of miR-19a-3p after 10 μM DEHP exposure could bring significant difference on gene expression then dramatic changes on cell apoptosis and primordial follicle assembly.

So we concluded that DEHP exposure increased ROS and oxidative stress responsive miRNAs, then influenced the key genes in the PI3K/AKT1/mTOR signaling pathway and induced cell apoptosis in the newborn mouse ovaries, thereby influencing cyst breakdown and primordial follicle assembly.

However, it's more complicated than that, because it's impossible for animals and human to be exposed to a single environmental chemical. The interactions between phthalates and other chemicals have been studied by combined exposure. The effects of combined exposure to multiple chemicals are not the superimposed effects of each. There are possibly synergistic or antagonistic actions among them (48, 49).

## Data Availability Statement

The datasets for this study can be found in NCBI under accession number GSE140323 https://www.ncbi.nlm.nih.gov/geo/query/acc.cgi?acc=GSE140323.

## Ethics Statement

The animal study was reviewed and approved by The Ethics Committee of Qingdao Agricultural University.

## Author Contributions

X-FS and WS designed the experiment. J-NZ and J-CL performed the experiments. R-QZ analyzed the data. LL drafted the manuscript. All authors read and approved the final manuscript.

### Conflict of Interest

The authors declare that the research was conducted in the absence of any commercial or financial relationships that could be construed as a potential conflict of interest.

## Supplementary Material

Figure S1 The detailed DEHP dilution flowchart.

## Abbreviations

DEHP: di (2-ethylhexyl) phthalate
MEHP: mono (2-ethylhexyl) phthalate
miRNA: micro-RNA
Dpc: days post-coitum
GO: gene ontology
MAPK: mitogen-activated protein kinase
mTOR: mammalian target of rapamycin
FoxO: forkhead box O
PTEN: phosphatase and tensin homolog deleted on chromosome ten
pAKT: Phosphorylated AKT
Bcl-2: B cell leukemia/lymphoma 2
Bax: BCL2 associated X
PI3K: phosphatidylinositol 3-kinase.
