# Supplementary figures and images for: Di (2-ethylhexyl) Phthalate Exposure Impairs the microRNAs Expression Profile During Primordial Follicle Assembly
Source: Front Endocrinol (Lausanne). 2019 Dec 13;10:877. doi: 10.3389/fendo.2019.00877 (PMC6923199; doi:10.3389/fendo.2019.00877)

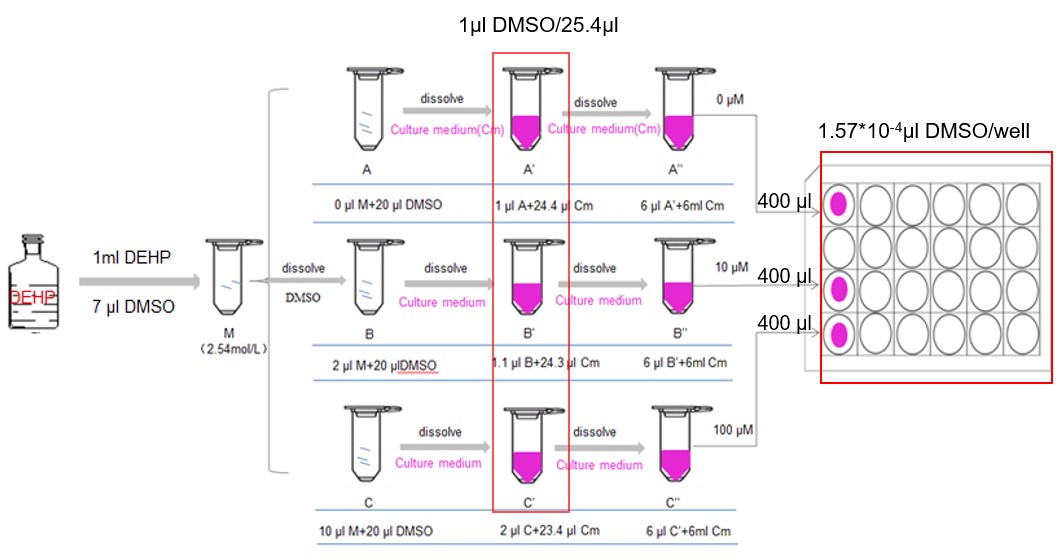

Supplement: Figure S1 — The detailed DEHP dilution flowchart. [file Image_1.JPEG]
